# Eye Tracking and Visualization: Introduction to the Special Thematic Issue of the Journal of Eye Movement Research

**DOI:** 10.16910/jemr.10.5.1

**Published:** 2018-05-25

**Authors:** Michael Burch, Lewis L. Chuang, Andrew Duchowski, Daniel Weiskopf, Rudolf Groner

**Affiliations:** Visualization Research Center, University of Stuttgart, Germany; Max Planck Institute for Biological Cybernetics, Tübingen, Germany; Clemson University,, USA; scians Ltd, Bern, and University of Bern,, Switzerland

**Keywords:** Eye movement, eye tracking, visualization, vision, cognition, perception, human-computer-interaction, usability, user experience

## Abstract

There is a growing interest in eye tracking technologies applied to support traditional visualization techniques like diagrams, charts, maps, or plots, either static, animated, or interactive ones. More complex data analyses are required to derive knowledge and meaning from the data. Eye tracking systems serve that purpose in combination with biological and computer vision, cognition, perception, visualization, human-computer-interaction, as well as usability and user experience research. The 10 articles collected in this thematic special issue provide interesting examples how sophisticated methods of data analysis and representation enable researchers to discover and describe fundamental spatio-temporal regularities in the data. The human visual system, supported by appropriate visualization tools, enables the human operator to solve complex tasks, like understanding and interpreting three-dimensional medical images, controlling air traffic by radar displays, supporting instrument flight tasks, or interacting with virtual realities. The development and application of new visualization techniques is of major importance for future technological progress.

There is a growing interest in eye tracking technologies, particularly applied to understand visualization techniques like diagrams, charts, maps, or plots, either static, animated, or interactive ones. However, in most cases the recorded eye movement data are too complex to be efficiently and fully explored with traditional visualization techniques, integrated into eye tracking systems like charts of frequency distributions or heat maps of perceptual and cognitive parameters. More complex data analyses are required to derive knowledge and meaning from the data, in particular to support the detection of design flaws, misinterpretations, and perceptual illusions in the stimuli, or to observe visual or attentional problems, all leading to degradations of performance at some tasks.

More advanced analyses can be based in two ways, either by algorithmic approaches trying to reduce the amount of data with the goal to find patterns, rules, and correlations, or by visualization approaches trying to exploit the strengths of the human visual system for rapidly detecting visual patterns. Combining algorithms and interactive visualizations with the user-in-the-loop is a powerful concept in the present days, referred to as visual analytics, with the goal of gaining knowledge from heterogeneous, conflicting, and big data, which is a reasonable description of eye movement data—to the behest of many eye movement researchers. “Computers are incredibly fast, accurate and stupid; humans are incredibly slow, inaccurate and brilliant; together they are powerful beyond imagination“ (a quotation attributed to Albert Einstein; however see the critique of Shoemaker, ( 13) ). Effective visualization techniques allow for complex information organization and re-organization, without compromising serendipitous discovery.

The immense flood of eye movement data has been made possible by the technological advances in computer vision algorithms combined with sophisticated sensor hardware that have become affordable to many researchers and research institutions all over the world. In fact, we are likely to witness even more complex eye movement datasets with the growing integration of eye trackers in consumer products as well as mobile eye-tracking in-the-wild ( 2 ). These advances make eye tracking applicable to several research fields like psychology, neuroscience, and optometry.

Eye movement data have an inherent spatio-temporal nature complemented by additional metrical and physiological data in a multivariate and dynamic form. Interpreting such complex and time-varying data together with the semantic meaning of the displayed stimuli consequently requires time-efficient algorithmic and heuristic concepts. To do this, the field of eye tracking and visualization has to take into account several related and interdependent fields like biological and computer vision, cognition, perception, visualization, human-computerinteraction, usability and user experience research. By the combination and interplay of those research disciplines, eye tracking becomes a powerful tool for evaluating stimuli like visualizations, and again using visualizations to find insights in the spatio-temporal domain of eye movement data.

This special issue is based on a follow-up workshop, parallel with the IEEE VIS Conference ( http://ieeevis.org ), Second Workshop on Eye Tracking and Visualization (ETVIS 2016) which took place in Baltimore, Maryland, USA, on October 23rd, 2016. For the special issue of the Journal of Eye Movement Research some authors who presented papers at the ETVIS 2016 workshop were invited for an extended version of their research as a full-length article. In addition, an open call for articles was made for interested researchers to submit an article that was not presented at the workshop. Following the normal peer reviewing process with 2-5 peer reviews according to the standards of the Journal of Eye Movement Research, some submissions were not accepted for publication. Those which had been revised and finally accepted by the editors of the special issue (who are also the authors of this introduction) were published on a rolling basis immediately after final acceptance in this special thematic issue.

In the article “A quality-centered analysis of eye tracking data in foveated rendering” the authors ( 10 ) present an analysis of eye tracking data for evaluating a foveated rendering approach for head-mounted displays. Foveated rendering methods adapt the image synthesis process to the user’s gaze, exploiting the capacities of the human visual system to increase rendering performance. Foveated rendering has great potential when certain requirements are fulfilled, like low-latency rendering to cope with high display refresh rates crucial for virtual reality at a high level of immersion.

The contribution “Gaze self-similarity plot - a new visualization technique“ ( 6 ) introduces a technique called gaze self-similarity plot that can be applied to visualize both spatial and temporal eye movement features on a single two-dimensional plot. The technique is an extension of the idea of recurrence plots, commonly used in time series analysis. The paper presents the basic concepts of the proposed approach together with some examples of what kind of information may be disclosed and shows some possible applications.

With the increasing number of studies where participants’ eye movements are tracked while watching videos, the volume of gaze data records is growing immensely. In “Visual analytics of gaze data with standard multimedia Player“ Schöning, Gundler, Heidemann, König, & Krumnack ( 11 ) define and utilize an exchange format that can be interpreted by standard multimedia players and can be streamed via Internet by using multimedia container formats for distributing and archiving eyetracking and gaze data bundled with the watched videos.

Several popular visualizations of gaze data, such as scanpaths ( 4 ) and heatmaps, can be used independently of the viewing task. For a specific task, such as reading, more informative visualizations can be created, as Špakov, Siirtola, Istance, & Räihä ( 12 ) demonstrate in “Visualizing the reading activity of people learning to read”. The authors present several static and dynamic techniques to communicate the reading activity of children to primary school teachers. Static visualizations help in getting a simple overview of how the children read as a group and of their active vocabulary, while dynamic visualizations help to give the teachers a good understanding of how the individual students read.

Although eye tracking data are in wide usage, little has been done to visually represent the uncertainty of recorded gaze data. In “Uncertainty visualization of gaze estimation to support operator controlled calibration“ Hassoumi, Peysakhovich & Hurter ( 5 ) demonstrate how visualization assets can support the qualitative evaluation of gaze estimation uncertainty. The authors’ gaze data processing method allows at every stage of the data transformation an estimate of uncertainty.

In the article “A skeleton-based approach to analyze and visualize oculomotor behavior when viewing animated characters” Le Naour and Bresciani ( 8 ) propose a new approach to quantify and visualize the oculomotor behavior of viewers watching the movements of animated characters in dynamic sequences. They illustrate the gaze distribution of one or several viewers by visualizing the timelines of the viewers by combining the spatial and temporal characteristics of the gaze pattern which provides an efficient tool to compare the oculomotor behaviors of different viewers.

In their study “Eye movement planning on single-sensorsingle-indicator displays is vulnerable to user anxiety and cognitive load“ Allsop, Gray, Bülthoff and Chuang ( 1 )demonstrate the effects of anxiety and cognitive load on eye movement planning in an instrument flight task on the basis of a single-sensor-single-indicator data visualization design philosophy. The task was performed in neutral and anxiety conditions, while at low or high cognitive load, auditory *n*-back task was performed. Higher cognitive load led to a reduction in the number of transitions between instruments and impaired task performance. The results suggest that both, cognitive load and anxiety, influence gaze behavior. These effects should be taken into account when designing data visualization displays.

In “Scanpath visualization and comparison using visual aggregation techniques“ Peysakhovich and Hurter ( 9 ) demonstrate the use of different visual aggregation techniques to obtain visual representations of scanpaths. Fixation points and saccades are aggregated using an algorithm that handles saccades direction, onset timestamp, magnitude or their combination for the edge compatibility criterion. Flow direction maps, computed during bundling, can be visualized separately or as a single image. The authors provide examples of basic patterns, visual search task, and art perception. Used together, the applied techniques provide new interesting information about the eye movement data.

Kumar, Netzel, Burch, Weiskopf and Mueller ( 7 ) in their article “Visual multi-metric grouping of eyetracking data“ present an algorithmic and visual grouping of eye-tracking data using two visualization concepts. First, parallel coordinates are used to provide an overview of the used metrics, their interactions, and similarities. Next, a similarity matrix is used to visually represent the affine combination of metrics. In an algorithmic grouping of subjects the eye-tracking data are encoded into the cells of a similarity matrix of participants, a procedure that leads to distinct visual groups of similar behavior. The authors illustrate this visualization by a data set of subjects reading metro maps.

In the article “Using simultaneous scanpath visualization to investigate the influence of visual behaviour on medical image interpretation“ Davies, Vigo, Harper and Jay ( 3 ) explore how a number of novel methods for visualizing and analyzing differences in eye-tracking data, including scanpath length, Levenshtein distance, and visual transition frequency, can help to reveal the methods clinicians use for interpreting electrocardiograms. Visualizing the differences between the scanpaths of the participants simultaneously gave answers to questions whether clinicians fixate randomly on the electrocardiograms or apply a systematic approach, and about the relationship between interpretation accuracy and visual behavior. Results indicate that practitioners have very different visual search strategies. Clinicians who incorrectly interpret the image have greater scanpath variability than those who correctly interpret it.

Taken together, the ten articles collected in this thematic special issue provide interesting examples how sophisticated methods of data analysis and representation enable researchers to discover and describe fundamental spatiotemporal regularities in the data. On the other hand, the human visual system, equipped with appropriate visualization tools, supports the human operator in complex tasks, like understanding and interpreting threedimensional medical images, controlling air traffic by radar displays, performing an instrument flight task, or interacting with virtual realities. The development and application of new visualization techniques is of major importance for future technological progress.

## Acknowledgement

The research of Daniel Weiskopf and Lewis Chuang is supported by the DFG SFB-TRR 161 (B01 & C03).
